# Mental health and help seeking behaviour in first year medical students

**DOI:** 10.1192/j.eurpsy.2021.2015

**Published:** 2021-08-13

**Authors:** V. Rutkauskas, J. Akavickas, A. Matuzaite, S. Lesinskiene

**Affiliations:** 1 Institute Of Health Sciences, Department Of Public Health, Vilnius University Faculty of Medicine, Vilnius, Lithuania; 2 Institute Of Clinical Medicine, Clinic Of Psychiatry, Vilnius University Faculty of Medicine, Vilnius, Lithuania; 3 Faculty Of Medicine, Vilnius University, Vilnius, Lithuania

**Keywords:** mental health, Medical Students, stress, mental health stigma

## Abstract

**Introduction:**

Mental health challenges are common among medical students. Data shows that that they are less likely to use mental health services, regardless of experiencing frequent mental health issues.

**Objectives:**

The aim of our study was to evaluate first year medical students’ mental health state and attitude to seeking help.

**Methods:**

The target group was the first year medical students in Vilnius University. Anonymous questionnaire created by authors was used to evaluate socio-demographic data, self-perceived emotional state level, attitudes and accessibility to mental health services. The study involved 152 first year medical students: 97 of them were local and 55 international students.

**Results:**

The majority of students (71.7%) reported that their studies negatively impacted their emotional condition. 14.5% of all students thought that they needed a consultation by mental health specialist, but decided not to seek help. 11.2% of students reported having used psychotropic drugs which had not been officially prescribed by a psychiatrist. 18.4% of all students thought that seeing a mental health specialist could negatively affect their future career as a doctor. 30.9% of students reported that they had used alcohol to improve their emotional state, 11.2% of students had used cannabis, 4.6% of students had used other drugs (e.g. LSD, amphetamine, cocaine) for this purpose.

**Conclusions:**

1. Majority first year medical students think that the begining of studies have negative impact on their emotional well-being. 2. A large number of medical students unwilling to see mental health specialist. 3. Significant number of students use psychoactive substances to improve their emotional state.

**Disclosure:**

No significant relationships.

